# Supplementary material for: Pollutant Gases to Algal Animal Feed: Impacts of Poultry House Exhaust Air on Amino Acid Profile of Algae
Source: Animals (Basel). 2024 Feb 28;14(5):754. doi: 10.3390/ani14050754 (PMC10931161; doi:10.3390/ani14050754)
Supplement: Supplementary file 1 [file animals-14-00754-s001.zip › animals-2826339-supplementary.pdf]

Table S1. The cost calculations of 1 L Bold's Basal Medium

Chemicals	The amount used for 1 L BBM (mg/Ld)	Price of each chemicals	Cost Turkish liras (TL)	Cost in Dollars
$KHPO_4$	175 mg/l	300 TL/1000 g	0.053	0.00654
$CaCl_2$*$2H_2O$	25 mg/l	115.15 TL/1000 g	0.003	0.00037
$MgSO_4$*$7H_2O$	75 mg/l	726.41 TL/1000 g	0.054	0.00667
$NaNO_3$	250 mg/l	751.22 TL/250 g	0.75	0.09259
K_2HPO_4	75 mg/l	253.45 TL/100 g	0.19	0.02346
MoO_3	1.42 mg/l	902.61 TL /100 g	0.013	0.00160
NaCL	25 mg/l	31.98 TL/1000 g	0.0008	0.00010
EDTA $C_{10}H_{16}N_2O_8$	50 mg/l	347 TL/500 g	0.035	0.00432
KOH	31 mg/l	103.62 TL/1000 g	0.0032	0.00040
$FeSO_4$*$7H_2O$	4.98 mg/l	142.87 TL/1000 g	0.0007	0.00009
H_2SO_4	1uL	130.41 TL/2500 mL	0.00005	0.00001
H_3BO_3	11.42 mg/l	179.42 TL/100 g	0.02	0.00247
Trace Metal Solution (1 mL/L)	1 mL	12.95 TL/1000 mL	0.013	0.00160
			Total cost for 1 L BBM	0.140216
